# Performance Consistency of AlSi10Mg Alloy Manufactured by Simulating Multi Laser Beam Selective Laser Melting (SLM): Microstructures and Mechanical Properties

**DOI:** 10.3390/ma11122354

**Published:** 2018-11-22

**Authors:** Bin Liu, Zezhou Kuai, Zhonghua Li, Jianbin Tong, Peikang Bai, Baoqiang Li, Yunfei Nie

**Affiliations:** 1 School of Materials Science and Engineering, North University of China, Taiyuan 030051, China; liubin3y@nuc.edu.cn (B.L.); kuaizezhou@163.com (Z.K.); baipeikang@nuc.edu.cn (P.B.); lbqlalala@gmail.com (B.L.); m18834046038@163.com (Y.N.); 2 School of Mechanical Engineering, North University of China, Taiyuan 030051, China; Evantjb@163.com

**Keywords:** AlSi10Mg, multi-laser manufacturing, selective laser melting, microstructure, mechanical property

## Abstract

Multi-laser beam selective laser melting (SLM) technology based on a powder bed has been used to manufacture AlSi10Mg samples. The AlSi10Mg alloy was used as research material to systematically study the performance consistency of both the laser overlap areas and the isolated areas of the multi-laser beam SLM manufactured parts. The microstructures and mechanical properties of all isolated and overlap processing areas were compared under optimized process parameters. It was discovered that there is a raised platform at the junction of the overlap areas and the isolated areas of the multi-laser SLM samples. The roughness is significantly reduced after two scans. However, the surface roughness of the samples is highest after four scans. As the number of laser scans increases, the relative density of the overlap areas of the samples improves, and there is no significant change in hardness. The tensile properties of the tensile samples are poor when the overlap area width is 0, 0.1, or 0.2 mm. When the widths of the overlap areas are equal to or greater than 0.3 mm, there is no significant difference in the tensile strength between the overlap and the isolated areas.

## 1. Introduction

Selective laser melting (SLM) is one of the most commonly used additive manufacturing (AM) technologies. SLM is a powder-based AM process which utilizes higher laser energy densities to facilitate complete melting and consolidation of successive layers of powder to fabricate 3D components [1,2]. Based on the idea of additive manufacturing, a three-dimensional CAD model is sliced into layers to obtain a 2D contour. Within the 2D contour, the laser selectively scans the metal powder layer and builds high-quality parts. It provides an almost unchallenged freedom of design, without the need for part-specific tooling [3,4,5]. Customized designs with complex internal and external structures using SLM technology are widely used in a variety of applications, including in automotive, aerospace, and biomedical industries [6]. 

AlSi10Mg is a typically-cast aluminium alloy that belongs to the Al-Si alloy group. AlSi10Mg alloy is a popular material in the aerospace and automotive industries due to its high specific strength and thermal conductivity [7,8]. In recent years, with the rapid development of additive manufacturing technology, rapid solidification technology has become the focus of research on grain refinement. 3D printing technology has become the research focus of Al-Si alloy manufacturing with SLM. It has unique advantages. Al-Si alloys fabricated by SLM could reach near full density under suitable processing conditions. Due to its rapid solidification with a high cooling rate, SLM Al-Si can exhibit ultrafine unstable and patterned microstructures, leading to some interesting properties, such as very high toughness and high strain-hardening ability. These results imply that SLM alloys might have quite different microstructures and performances compared with materials fabricated by conventional ingot metallurgy or powder metallurgy routes. Some research institutes, both foreign and domestic, have studied the laser-forming technologies of aluminium alloys [9,10]. At present, the SLM manufacturing of Al-Si-based AlSi10Mg has been studied abroad, including research on single-channel, single-layer manufacturing quality [11], the relationship between process parameters and moulding quality, process interval optimization [12,13], characterisation, control of microstructures [14,15,16,17], and microstructure and mechanical properties [18,19,20].

With low building efficiency, most SLM devices currently have the disadvantage of being unable to fabricate large-sized parts. Currently, the most effective way to improve the building efficiency is to increase the scanning speed and the thickness of the powder layer under high laser power. However, improving the laser power causes the laser spot diameter to increase, which may affect the accuracy and therefore the physical and mechanical properties of the part. Multi-laser beam machining is an effective way to increase the size and efficiency of SLM-shaped parts. Multi-laser SLM technology can effectively increase the build rate, reduce manufacturing time, and produce larger parts. In laser manufacturing, each independent but synchronizable laser is responsible for manufacturing different areas. The manufacture time can be greatly reduced and can further expand the forming space. In this way, multiple lasers will form an important development regarding the direction of SLM. Wang Zemin et al. [21] studied the microstructure and mechanical properties of the four-laser fabricated Ti6Al4V overlap areas and isolated areas using self-developed multi-laser equipment. The results illustrated that the stair-step phenomenon of the overlap areas can be eliminated by the compartment rotation scan, and also there was no significant change in the microstructure or mechanical properties of the overlap areas. Multi-laser SLM is feasible for fabrication of large size Ti6Al4V parts. Andreas Wiesner et al. [22] used the SLM 500HL multi-laser device to produce large cylinder heads, each with dimensions 494 × 210 × 143 mm. The manufacturing time was reduced from 170 h for dual lasers to 85 h for four lasers, and the production efficiency was greatly improved. This both highlights an important breakthrough in multi-laser SLM additive manufacturing and proves that multi-laser SLM manufacturing has significant advantages. Buchbinder D et al. [23] used a new prototype machine tool including a 1 kW laser and a multi-beam system to improve the efficiency of aluminum. The results of the investigation demonstrated that the build rate for the production of AlSi10Mg parts can be increased by using a 1 kW laser. There are still many other researchers who have studied multi-laser manufacturing technology. Thorsten Heeling et al. [24,25] studied the effect of the laser compensation process on the stability of SLM formation. This study discovered that the two lasers work together with the melt pool and the proper power compensation, and focus positioning of the second laser can more accurately control the laser melting process. This helps to stabilize the melt pool, obtain dense parts, reduce temperature gradients, and reduce residual stress. Weingarten et al. [26] used just one beam, but they scanned the part two times to dry the aluminium powder bed in the process, prior to melting, to reduce the amount of hydrogen porosity. Abe et al. [27] used a second moving beam in a simple test to selectively reduce the cooling rates and thus influence the microstructure with promising results.

In summary, leading SLM equipment manufacturers are competing to develop their own multi-laser SLM equipment. However, there are few reports on multi-laser SLM manufactured parts. There are few public reports on the performance consistency of multi-laser SLM manufacturing Al alloys. Here we will study the changes of macroscopic morphology, phase distribution, and mechanical properties of the double-laser overlap area, the four-laser overlap area, and the isolated area. This will include analyzing the change mechanism to promote the laser SLM technology and theoretical and technical support in the extensive and in-depth application of shaped aluminium alloy components.

## 2. Experimental Methods

**Figure 1:**
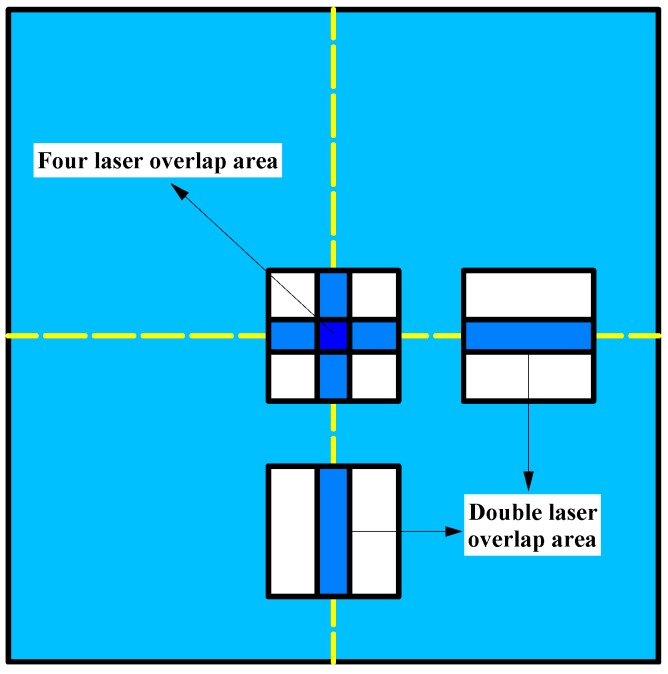
Laser bonding area schematic.

**Figure 2:**
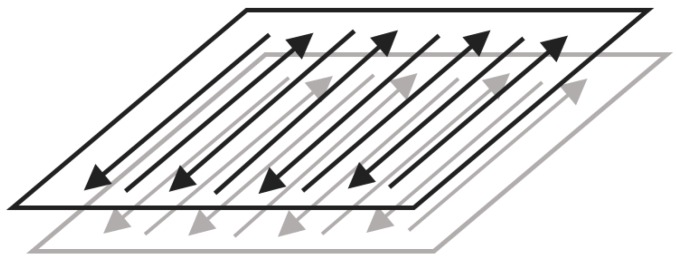
Selective laser melting (SLM) scan direction.

**Figure 3:**
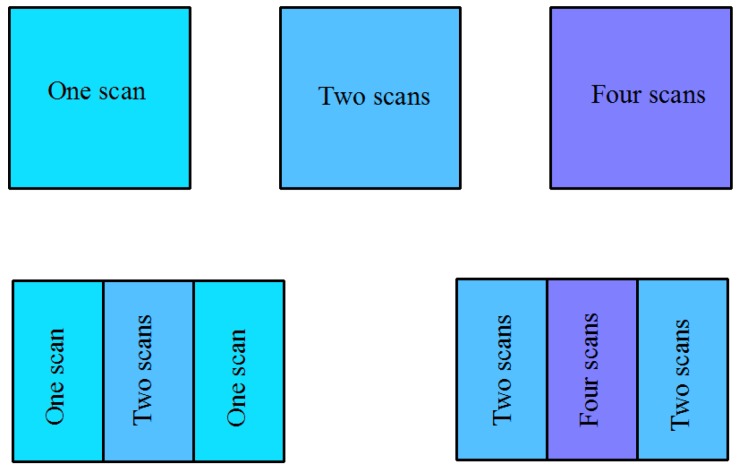
Experimental design of multi-laser manufacturing.

Multi-laser manufacturing is achieved by simulation of a single laser device (Renishaw AM-400, Renishaw, Gloucestershire, England). The machine is equipped with a modulated ytterbium fiber laser with a wavelength of 1070 nm. This project uses Quant AM software (2016, Renishaw, Gloucestershire, England) to splice two or four parts, and the overlapping area is repeatedly melted under the set process to achieve multi-laser bonding, as shown in Figure 1. Also, the optimized processing parameters employed are listed in Table 1, and the scanning direction is shown in Figure 2. The double-laser overlap area is equivalent to the laser repeating the scanning of one area two times, and the same four-laser overlap area is equivalent to repeating the scanning four times. A single-laser device (Renishaw AM-400) scans one, two, and four times per layer to achieve single laser, double-laser, and four-laser formed parts, respectively. The sizes of the samples are 10 (L) × 10 (W) × 10 (H) mm, and the other two formed samples have sizes of 15 (L) × 10 (W) × 10 (H) mm. The area with an intermediate size of 5 (L) × 10 (W) mm is a double-laser and a four-laser overlapping area. The experimental design of multi-laser manufacturing is shown in Figure 3.

**Figure 4:**
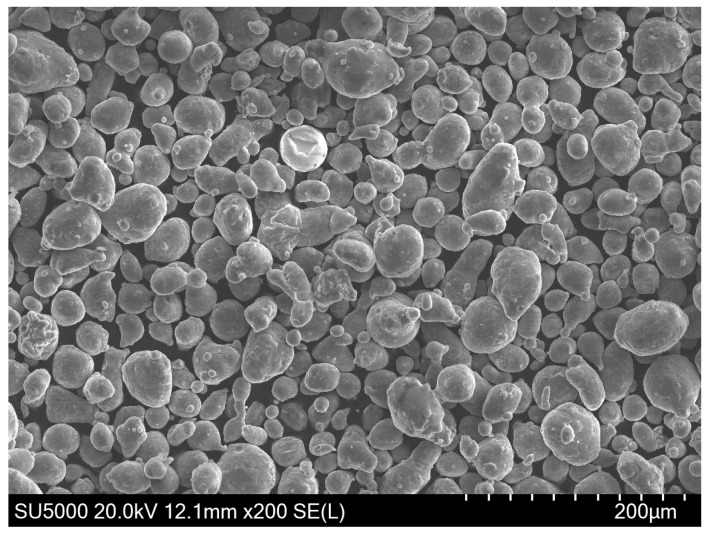
SEM of AlSi10Mg powders.

**Figure 5:**
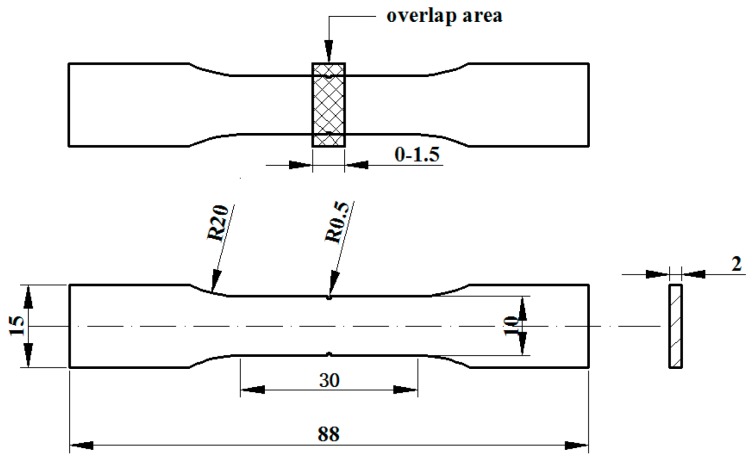
Standard dimension drawing of a tensile specimen (mm).

The AlSi10Mg powder used in this study was provided by LPW Technology with the chemical element composition of the AlSi10Mg powder, as shown in Table 2. In the AlSi10Mg powder alloy, the Al element is a matrix material and the main alloying element is Si. The powder is prepared by gas atomization and has a spherical shape. The average particle size is 45 μm, obeying normal distribution and having good flowability, and its powder electron microscope scanning (SEM, SU5000, HITACHI, Tokyo, Japan) topography is shown in Figure 4. Tensile samples in isolated areas and overlap areas were built according to Figure 5 in this experiment. The overlap width of the tensile samples is 0–1.5 mm. The overlap areas of the tensile samples were scanned twice.

The surface roughness was measured using the surface roughness step measuring instrument (JB-4C, Taiming Optical Instrument Co., Ltd, Shanghai, China). Metallographic observations were made using a polarising microscope (Axio Scope A1, Carl Zeiss, Heidenheim, Germany) and the phase identification was identified through an X’Pert-Pro X-ray diffractometer (D/max-rB, Rigaku Corporation, Tokyo, Japan). The hardness values were tested (11 times on each type of sample) using a digital micro Vickers hardness tester (TMHVS-1000, Yuming Optical Instrument Co., Ltd, Shanghai, China). Tensile properties of this experiment were tested using a microcomputer-controlled electronic universal testing machine (CMT5105, MTS industrial systems (China) co. LTD, Shenzhen, China). The number of the tensile tests carried out on each type of sample was one. The tensile rate has been used for carrying out the tensile tests of 2 mm/min. Following testing, the fracture surface of the tensile sample was characterized using a scanning electron microscope (SU5000, HITACHI, Tokyo, Japan).

## 3. Results and Discussion

### 3.1. Surface topography and Microstructural Analysis

#### 3.1.1. Overlap Boundary Effect

**Figure 6:**
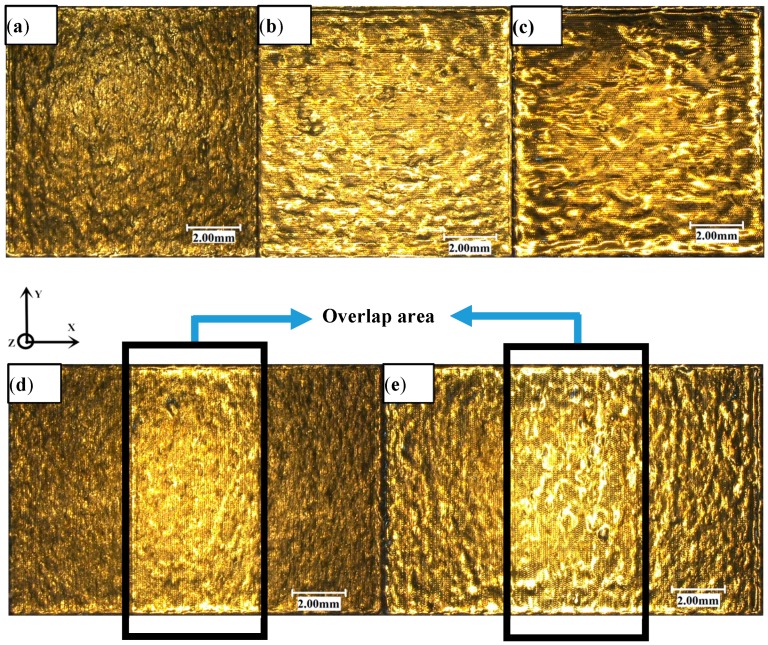
3D super-depth analysis of field microscopic surface topography at (**a**) one scan, (**b**) two scans, (**c**) four scans, (**d**) overlap—two scans, and (**e**) overlap—four scans.

The five images shown in Figure 6 are the super-depth maps of the upper surface of the sample using different remelting times and lap joints under the same process parameters. Figure 6a shows the laser was scanned only once throughout the area (i.e., metal powder only melts–solidifies once). Figure 6b shows the laser was scanned twice in the area, and Figure 6c shows the laser was scanned four times in the area. Figure 6d shows the lap area is laser scanned twice (often called remelting), while the isolated area is scanned only once. Figure 6e shows the lap area was scanned four times, while the isolated area was scanned twice. The areas indicated by the black line frames in Figure 6d,e are the overlap areas.

**Figure 7:**
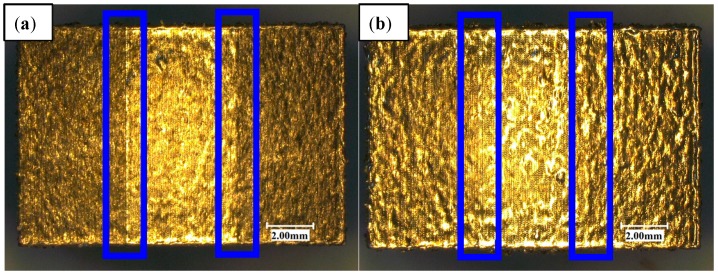

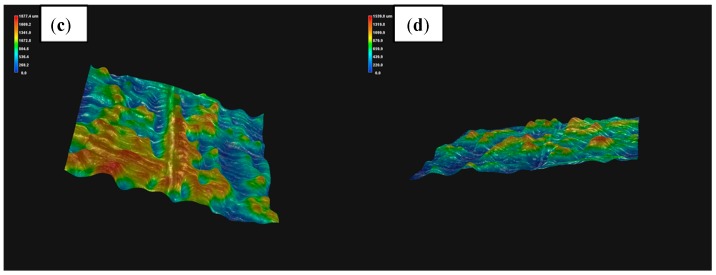
The stair-step effect of overlap area (**a**), overlap—two scans (**b**) overlap—four scans (**c**), and (**d**) three-dimensional contours of (**a**,**b**), which are marked blue.

Figure 6a,b shows the surface topography of a single-layer scan of AlSi10Mg powder after one and two scans. It can be seen that the surface topography after two scans is better than the un-remelting surface topography; not only is the oxide small, but also the surface is relatively clean. The overlap between the molten pools is also improved. Therefore, the single layer after two scans is more conducive to the lower layer connection than the single layer scanned one time so that the layers are more densely bonded together. By observing the three-dimensional microscopic surface topography of the upper surface of the sample, we can see that there is a unique phenomenon in the overlap and the isolated areas, which we call the overlap boundary effect. The overlap area has a distinct convex phenomenon compared to the isolated area (the blue line frame in Figure 7). Figure 7c,d show the three-dimensional contours of samples marked in Figure 7a,b for a more intuitive observation. This phenomenon is mainly due to the fact that the laser melts the metal powder and produces a solid metal layer following the first scan. When the laser scans the overlap area again, the metal powder that is not completely melted absorbs energy, subsequently generating a new melt pool. The molten metal pool solidifies again and generates a new metal layer. The two metal layers are then superimposed on the overlap areas so that the overlap area is increased in the *Z*-direction dimension when compared with the isolated area, resulting in a convex phenomenon. The bath temperature at the boundary is lower than the central temperature. The viscosity of the molten metal is high and the fluidity is poor, so the phenomenon is more obvious.

Through visual observation we found that with Figure 7b (four scans in the overlap area and two scans in the isolated area), when compared to Figure 7a (two scans in the overlap area and one scan in the isolated area), the overlap boundary effect has a weakening tendency due to the fact that the laser scans the solid metal layer into a liquid state. The liquid metal has improved fluidity, and therefore the molten metal flows to the isolated area and fills the pits in the isolated area, thereby relieving the generation of the overlap boundary effect. Therefore, increasing the number of scans can effectively reduce the overlap boundary effect.

#### 3.1.2. Surface Roughness

**Figure 8:**
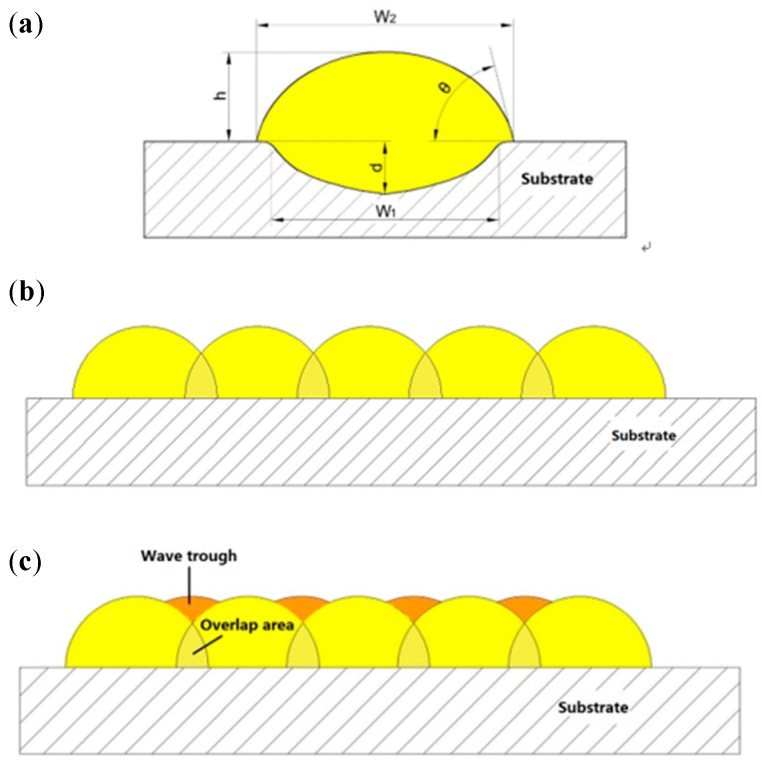
(**a**) Cross-section of the melting channel (W_1_ is the penetration area width, W_2_ is the width of the melting track, h is the height of the melting channel, d is the penetration depth, and θ is the wetting angle); (**b**) overlap schematic melting channels; (**c**) schematic diagram of the melting channel overlap area.

The roughness of the samples mainly includes the upper surface roughness and the side roughness. This paper only studies the surface roughness of multi-laser SLM manufacturing parts. The surface roughness of the part is mainly related to the quality of the manufacturing surface of a single melting channel (the cross-sectional view as shown in Figure 8a). We analyze the splicing process of the SLM melting channel (Figure 8b). Since the melting channel is elliptical in the manufacturing process, the melting channels cannot overlap completely, thereby causing a wave-like distribution between the two melting channels. Finally, the surface roughness is increased. A better way to improve the surface roughness caused by this factor is to properly control the scanning spacing (i.e., the reasonable overlap ratio). Reasonable scanning spacing can press the molten metal between the two melting channels to the trough area, which can effectively reduce the height difference of the trough area and reduce the surface roughness of the parts (the principle is shown in Figure 8c). The optimal situation is when the trough area formed by the tangent and arc of the two melting arcs is equal to the area of the overlapping area. Under such theoretical conditions, the surface roughness of the formed part is the smallest.

There are many factors affecting the surface roughness of the parts, including the parameters of the manufacturing process, the movement accuracy of the manufacturing equipment, the particle size distribution of the metal powder material, the sealing of the forming cavity, and the purity of the inert gas. Different parameters in the forming process often have different effects on the roughness of the parts. In view of the above, we only change the number of laser scanning metal powders under the condition that other process parameters are consistent.

**Figure 9:**
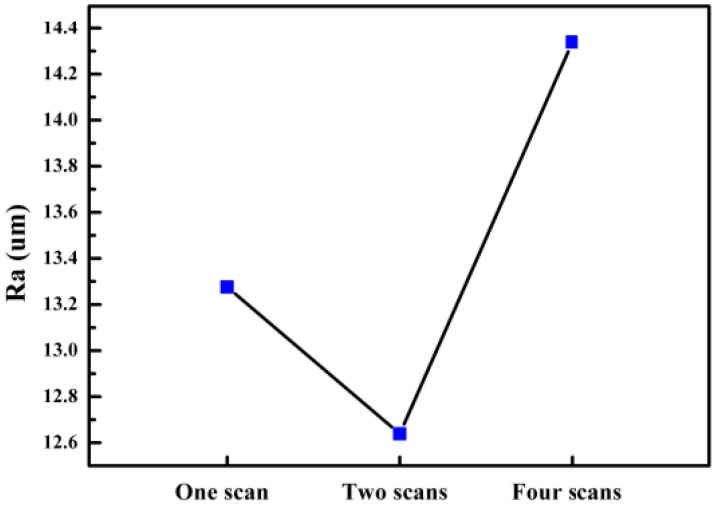
The effect of the number of scans on surface roughness.

According to the data shown in Figure 9, it can be concluded that the average Ra of the upper surface of the sample after one scan is 13.276 μm and after two scans is 12.639 μm. As shown, there is a significant drop, and the surface quality of each area is greatly improved. However, the average Ra of the upper surface of the sample after four scans is 14.339 μm.

The sample with a single laser scan has different particle sizes of metal powder. When the laser functions, the large-particle metal powder, i.e., the surface of the powder particles, melts. However, the inside of the powder cannot be melted due to insufficient energy. The metal powder does not undergo a complete melting-solidification process, although a small particle ball is formed inside. In the metal powder, there are often some powders with smaller particles that cannot be completely melted, and a phenomenon of adhesion occurs between these completely melted metal powders and unmelted metal powders, which results in small particle spheroidisation. SLM is a process in which a layer of metal is superimposed. Since the upper metal surface is not ideal, there are “convex peaks” and “pits” where the thickness of the powder layer is low at the “convex peak” and the thickness of the powder layer at the “pit” is high after the powdering roller is laid. After the laser scan, the metal powder at the thicker powder layer cannot be completely melted, and the molten metal adsorbs the unmelted metal particles outside the molten metal during the solidification process, forming a discontinuous melting channel and increasing the surface roughness. After understanding the melting channel and the formation process of the overlap area (Figure 8), it was determined that one of the factors causing the surface roughness of the upper surface of the laser scanning sample was the scanning direction. Figure 8 shows that one of the factors causing the surface roughness of the upper surface of the laser scanning sample is the scanning direction. The scanning direction of the experimental sample is one-way scanning, that is, the laser scans each layer of metal powder according to a fixed scanning path. The scanning path is the highest position of the melting channel, and the fixed scanning path superimposes the melting channel so that the scanning path becomes increasingly higher, and the wave troughs formed in the middle of the two scanning lines get increasingly lower. 

The remelting process (which occurs during double laser scanning) produces smoother, more uniform layers, and efficiently reduces the number of pores formed between the neighboring melt pools at the scan track edges [28,29]. Previous data analysis shows that the surface roughness of the sample after two laser scans can be effectively improved. In the sample scanned by one laser, some of the metal powder particles could not be completely melted, i.e., some of the particles remained in the powder state, which made the sample spheroidised. When the laser was scanned again, these powders could be completely melted to produce a new molten metal which was finally cooled and solidified to produce a metallurgical bond. The spheroidisation of the particles improved, effectively reducing the surface roughness. Due to the scanning direction, the highest point of the melting channel becomes increasingly higher. When the laser is scanned again, the fluidity of the molten metal in the melt pool is increased so that the molten metal originally at the highest point of the melting channel flows towards the “pit”, which flattens and reduces the peak between the two melting channels. At the height difference of the valley, the unevenness is alleviated, the surface roughness value is lowered after solidification, and the surface quality is improved.

When the sample is subjected to four scans, the surface roughness value increases and the molten metal is excessively melted, resulting in over-burning. This excessive melting reduces the viscosity of the molten metal, increases the wetting angle of the melt pool, and easily forms a spherical metal liquid, which causes spheroidisation defects as well as discontinuity of the melting channel and increased surface roughness.

#### 3.1.3. Microstructural Analysis

**Figure 10:**
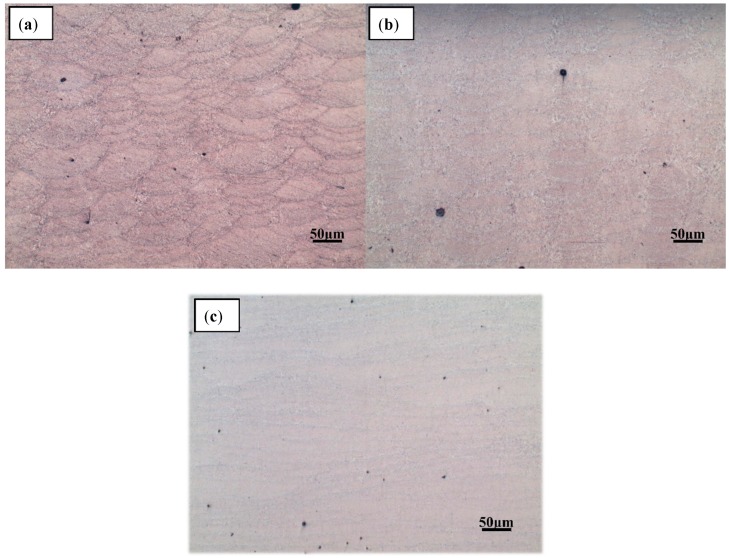
Optical micrograph of the left side of a multi-laser SLM sample (200 times) after (**a**) one scan, (**b**) two scans, and (**c**) four scans.

**Figure 11:**
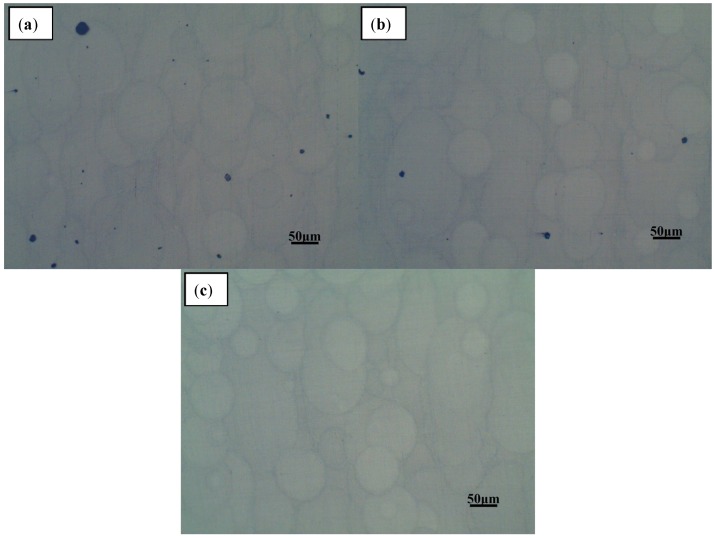
Optical micrograph of the upper surface of multi-laser SLM samples after (**a**) one scan, (**b**) two scans, and (**c**) four scans.

The AlSi10Mg sample was removed from the substrate using a wire cutter, and both the upper surface of the sample and the left side were polished and etched. The processed samples were observed under a metallographic microscope where the microstructure was observed. The left side is shown in Figure 10, and the upper surface is shown in Figure 11. 

The microstructure illustrated in Figure 10 shows a dendritic solidification similar to those observed in other studies [14,30]. The brighter areas are aluminium solid solutions, and the darker are silicon or Al-Si-eutectics. Generally, SLM samples show a finer microstructure than, for example, die-cast parts, due to the faster solidification. By observing the shape of the melt pool in Figure 10 and Figure 11, there is no splitting between the neatly arranged melting channels. The overlapping effect between the melting channels is clear, and the pores are relatively small. By comparing the pores in the melt pool under different laser scanning times, it can be found that there are many internal pores—some large—during a single laser scan. When the number of laser scans is increased once, the pores inside the part are significantly reduced. When the number of scans is increased to four, it is found that the internal pores are almost the same as with two scans, and most of the pores are small and rounded. The internal pores are mainly concentrated on the boundary and are mostly rounded. By observing the lap joint of the melted channel, a relatively obvious weld pool boundary can be seen after one laser scan. However, as the number of scans increases, the boundary of the melt pool becomes less obvious, and the higher the number, the less obvious it becomes.

There are two main reasons for the laser to produce a single scan: (1) The internal gas cannot escape completely, and (2) because of the spheroidise effect. When observing the SEM image of the metal powder, the metal powder is mostly spherical. The distance between the valleys of the two melting channels is too small, and the spherical metal powder cannot completely fill the area. When the laser scans these areas, the existing gaps in that area become the internal pores. The reason for the pores may also be due to the inability of the inert protective gas to escape during the solidification of the metal. Although some gaps can be filled with metal powder, others are relatively deep and, therefore, the laser powder cannot penetrate completely and the metal powder is difficult to completely melt. The molten metal liquid encloses the unmelted powder particles and may form large spheroidised particles after solidification, which leads to the generation of pores. Some of the unmelted metal powder is adsorbed around the melt pool to form spheroidised particles, which causes the melting channels to not be completely connected, which is why most of the pores exist in the boundary region with the melting channels. 

As the laser scans again, the spheroidisation caused by the previous laser scan can be attenuated. The laser scanning, again, can improve the fluidity of the molten metal which fills the area where the metal powder is difficult to fill and can reduce the generation of voids. According to the analysis of 3.1.1, laser scanning again can reduce the surface roughness of the upper metal layer so that the metal powder existing between the melting channels is reduced, which further reduces the presence of deeper gaps. In this way, the unmelted powder of the deep gap can also be reduced, and the number of pores in the parts can be reduced. This also reduces the porosity inside the part. Laser remelting melts the previously unmelted powder, reducing spheroidisation caused by unmelted metal powder and reducing voids inside the part.

The metal powder is completely melted after four rounds of laser scanning, resulting in a fluid metal liquid. However, during the scanning process, the surface of the melt pool will splash small metal droplets and form small spheroidized particles after rapid cooling and solidification. This explains the more minor pores, as shown in Figure 10c. Part of the reason is that multiple laser scanning can make the molten metal absorb more energy, and the molten metal is vaporized. As the energy elapses, the molten metal vaporizes and then solidifies to form a small spheroidisation phenomenon and subsequently generates pores. 

### 3.2. Phase Distribution

**Figure 12:**
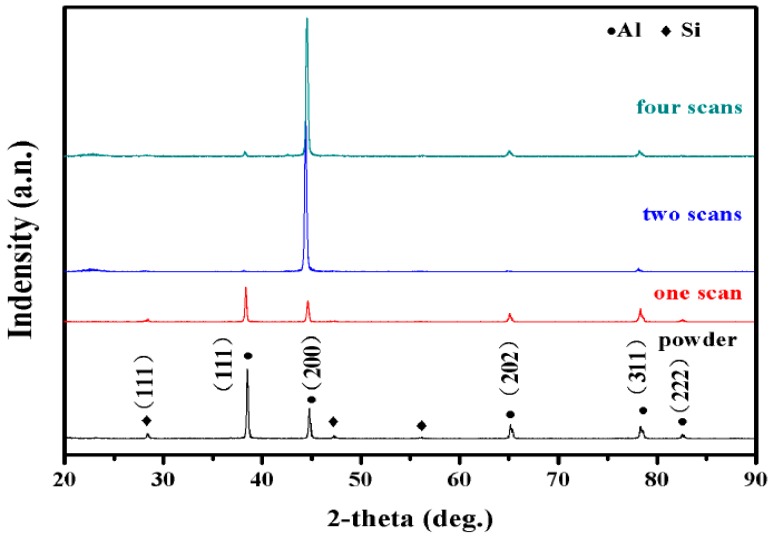
XRD patterns of AiSi10Mg powders and samples.

XRD patterns (Figure 12) show that the phase composition of a-Al and Si are found both in AiSi10Mg powders and AlSi10Mg samples (i.e., the microstructure was mainly composed of an Al/Si phase). It can be seen from the spectrum in the XRD that the peak of Al in the SLM sample and the peak corresponding to the powder are shifted to the right. As the number of laser scans increases, the rate of solidification increases. As a result, Si formed a solid solution in the Al matrix, and failed to precipitate in the form of primary silicon and eutectic silicon. This results in a decrease in the peak of Si and an increase in the peak of a-Al. The preferred orientation shown is the (200) plane. This is related to the microstructure and texture characteristics of AlSi10Mg as produced by the SLM process.

### 3.3. Mechanical Properties

#### 3.3.1. Density Measurements

**Figure 13:**
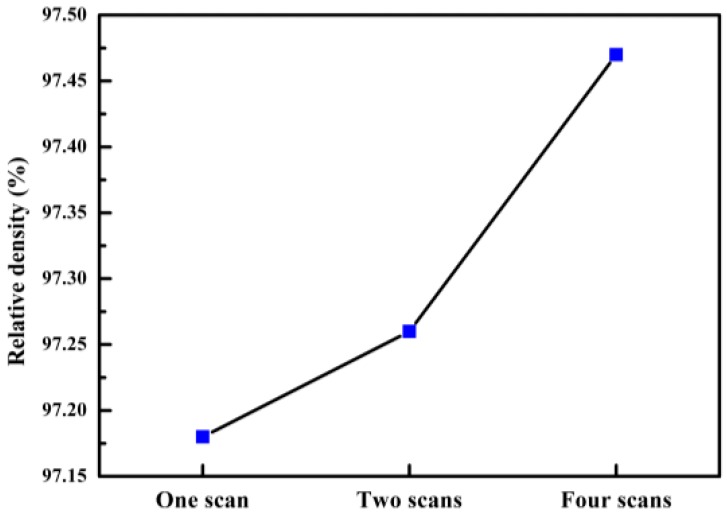
The relative density of different scan times.

The experimental scheme adopts the drainage method, and the standard density of the material is 2.68. The experimental data is shown in Figure 13. Through experimental data analysis, although the number of scans is different, the relative density of the sample is maintained above 97%. As shown in Figure 13, there are some trends in the number of scans, the number of scan increases, and the relative density increases.

Variations in the thermo-kinetics and thermo-capillary characteristics within the melt pool, such as the viscosity, wettability, and liquid-solid rheological properties, all influenced the densification behavior [31]. The relative density of the part has an important relationship to the presence of internal pores. When there are more internal pores, the relative density of the parts will decrease, and vice versa. When the laser is scanned once, laser scanning fails to completely melt the metal powder, which is prone to spheroidisation, resulting in some very large holes around the melting channel; i.e., the relative density of the parts is decreased. After the laser scans twice, the internal unmelted powder is scanned again by the laser, which can reduce the porosity generated by the unmelted powder and increase the relative density of the parts. By increasing the number of laser scans, the metal powder is fully melted, and the melt pool is fully spread and infiltrated. Therefore, the pores generated in the upper layer are filled, which contributes to the improvement of the internal structure to increase the relative density of the parts. The increase of laser scanning times will increase the relative density of parts. However, there is no significant change in the relative density.

#### 3.3.2. Microhardness Tests

**Figure 14:**
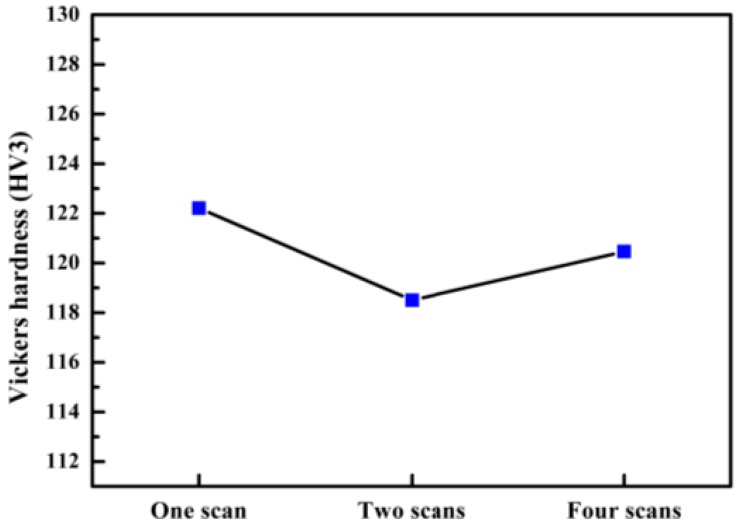
Vickers hardness of different scan times.

The analytical hardness test data were processed with the analysis results shown in Figure 14. We have obtained the average Vickers hardness of each sample, as shown in the bar graph. According to the analysis, the Vickers hardness of the sample scanned by one laser is greater than that of the sample scanned twice and four times, and the difference between the highest point and the lowest point is more than ten. The difference between the other two samples is controlled within ten. By comparing the average Vickers hardness, it can be found that the hardness of the sample after laser remelting decreases, but the change is not obvious and the difference is below five.

Samples that are scanned only once by the laser may feature defects such as voids, spheroidisation, and inclusions due to partial melting of the metal powder, which reduces the microhardness of the material. Some of the defects contained in the area are less rigid and some areas may have larger pores and other defects, causing a decrease in hardness which causes the hardness data to have a larger amplitude. When the laser scans the sample multiple times, the grains and the microstructure become larger. According to the Hall-Petch theory [32,33], grain refinement increases the number of crystal grains contained in the same volume, and internal dislocations reduce this number. When the part is subjected to an external force, the deformation occurs between a large number of crystal grains, and the slip of the dislocation is blocked by the grain boundary so that the expansion cannot be continued, thereby achieving fine grain strengthening. Such grain refinement increases resistance against external forces and increases the hardness.

Although the Vickers hardness of the sample scanned by the laser is decreased, the hardness variation is not particularly remarkable, so the microhardness of the multi-laser overlap area is the same as the isolated area.

#### 3.3.3. Tensile Behavior

In several studies, the mechanical properties of SLM manufacturing parts are affected by the internal melting channel structure, pores, cracks, residual stresses, inclusion defects, etc. The smaller the internal porosity, the smaller the residual stress, and the higher the density, the better the mechanical properties. The influence of these defects is mainly related to the SLM manufacturing process parameters, and the different scanning directions will also result in a structurally different internal melting channel. Kempen K et al. [11] conducted an experimental study on the mechanical properties of SLM-manufactured AlSi10Mg alloy. The results showed that the AlSi10Mg parts manufactured by SLM have improved or equivalent mechanical properties as compared to the cast AlSi10Mg materials. The Vickers hardness of the prepared SLM part is much higher than that of the high-pressure casting (HPDC) AlSi10Mg alloy, and the ultimate tensile strength is always higher than that of the AlSi10Mg alloy under HPDC. In this experiment, we mainly studied the tensile strengths of the overlap areas of two laser scans and the influence of overlap areas of different sizes on tensile properties.

**Figure 15:**
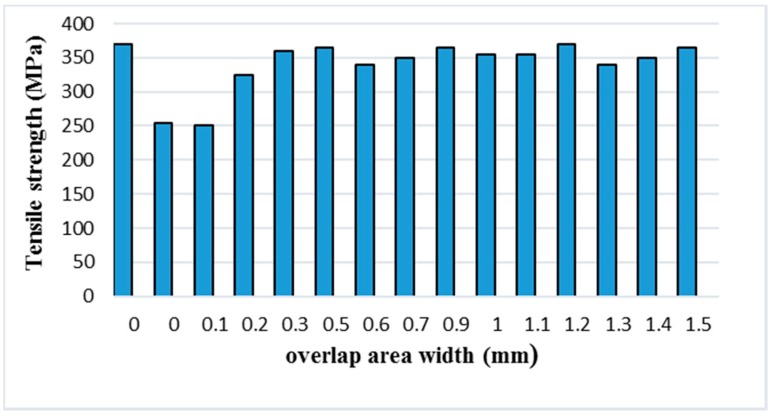
The tensile strength of tensile samples with overlapping areas of different sizes.

Currently, the SLM-manufactured parts cannot achieve the dimensional accuracy of the tensile samples. There are some small powder bonding particles on the forming surface, and the surface roughness is significant. There was also an error in the wire cutting machine when the samples were removed from the substrate. Taking into account the above factors, we increased the thickness of the samples to 2.5 mm. Test one measures the tensile strength of the standard piece of one scan. The other samples were twice-scanned with different overlap widths. The experimental data was then analyzed and processed, and the experimental results are shown in Table 3 and Figure 15. It can be seen from the table that the tensile strength of the tensile samples is much lower than that of the standard samples when the width of the overlap area is 0, 0.1, or 0.2 mm, and becomes larger as the overlapping width increases. The width of the overlap areas is small so that the powder of the overlap portion is not sufficiently melt-bonded to cause a decrease in tensile strength. When the widths of the overlap areas are equal to or greater than 0.3 mm, the tensile strengths of the samples are remarkably improved. However, the tensile strength decreased when compared with the standard samples, and the change was not clear. The mechanical properties of the parts are related to the hardness. The tensile strength and the microhardness are analyzed simultaneously. It was found that the number of laser scans has the same trend against the tensile strength and microhardness, whereas when the microhardness of the part increases the tensile strength also increases. There are many pores inside the sample that the laser scanned only once, and the increase of porosity will reduce the tensile strength and mechanical properties. Multiple laser scanning will also make the tissue coarse. According to the Hall-Petch theory, both the part’s performance and tensile strength will decrease.

**Figure 16:**
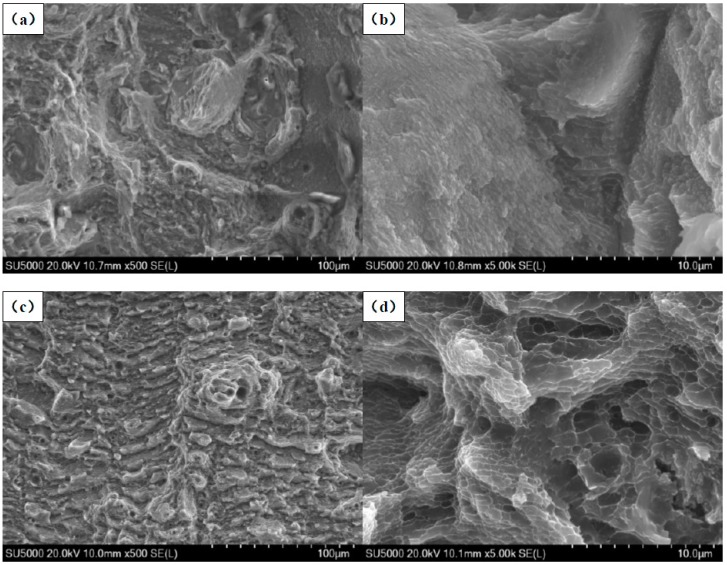
Fractographs of AlSi10Mg tensile samples at different scan times: (**a**) Low and (**b**) high magnification of one-scan SLMed samples; (**c**) low and (**d**) high magnification of two-scan SLMed samples at a high magnification.

In order to further understand the influence of the multi-laser process on the properties of SLM AlSi10Mg alloy, the fracture morphology of SLM AlSi10Mg alloy samples under different scanning times was analyzed. Figure 16a,b show the fracture morphology of the standard samples at one scan. Figure 16c,d show the fracture morphology of the samples at two scans. The overlap area of the sample in Figure 16c,d has a width of 1 mm. It can be seen from the figure that the SLM AlSi10Mg alloy has apparent dimples at the fracture at one scan, which is a typical ductile fracture. 

As can be seen in Figure 16c, the SLM AlSi10Mg alloy demonstrates smaller dimples. Additionally, the dimple zone on its fracture surface is smaller than that of the SLM AlSi10Mg alloy at two scans in Figure 16a. The formation of dimples implies a ductile rupture, whereas the smooth areas show cleavage facets in the fractured surface indicating a brittle fracture [34]. During the two scans, the number of dimples at the fracture was reduced, and the smooth brittle fracture surface increased. It can be seen from Figure 16b,d that the microstructures become large after two scans, and the internal pores increase, thus the performance of the part will decrease.

In summary, an increase in the number of laser scans will slightly reduce the mechanical properties of the part, but this change is less apparent. Therefore, the overlap areas of the multi-laser SLM samples have no significant change in tensile properties compared to the isolated areas. 

## 4. Conclusions

In this study, the multi-laser beam SLM of AlSi10Mg has been carried out to reveal its microstructure and mechanical properties. The overlap area and isolated area of the multi-laser SLM-manufactured part are systematically studied. The main results can be summarised as follows:

1. The morphology and microstructure of the melt pool of the parts with different scanning times were analyzed. The analysis established that the melting channels were arranged neatly, the overlap boundary effect between the melting channels was clear, and the pores were relatively small. Comparing the internal pores of the part, the inside of the laser scan contains some unmelted metal powder, which causes more pores. Following laser remelting, the pores are reduced, and only some tiny pores exist. After four laser scans, micro-spheroidised particles are produced, and the internal pores are formed as small round hole shapes.
2. The number of laser scans was used as the research object to investigate the influence of the number of scans on the surface quality of the parts. It was found that there is a bulge at the junction of the overlap area and the isolated area of the multi-laser SLM manufactured part, which is called the overlap boundary effect. The average Ra of the upper surface of the sample after one scan is 13.276 μm, and the average Ra of the surface of the sample after two scans is 12.639 μm. However, the average Ra of the upper surface of the sample after four scans is 14.339 μm. A laser scan will result in spheroidisation of the unmelted powder and will increase the surface roughness of the part. Two laser scans can improve this situation and reduce surface roughness. However, increasing the number of scans may lead to spheroidisation and increased surface roughness.
3. It can be seen from the spectrum in XRD that the peak of Al in the SLM sample and the peak corresponding to the powder are shifted to the right. As the number of laser scans increases, the preferential crystallite growth orientation reaches the (200) plane.
4. The relative densities of the multi-laser SLM samples are above 97%, and the increase in the number of laser scans increases the relative density. The microhardness of the samples with different scanning times did not change significantly. The tensile properties of the multi-laser manufactured parts were examined by fabricating tensile specimens of overlap areas with different widths. When the width of the overlap areas is 0, 0.1, or 0.2 mm, the tensile strength of the tensile samples is much lower than that of the standard samples. When the width of the overlap areas is equal to or greater than 0.3 mm, the tensile strengths of the tensile samples are slightly lower than the standard samples.

## Figures and Tables

**Table 1:** SLM process parameters used for manufacturing AlSi10Mg samples.

Manufacturing Parameters	Value
Laser power, P	200 W
Scan space, S	80 μm
Powder layer thickness, h	25 μm
Spot diameter, D	80 μm
Exposure Time, ET	140 μs
Point Distance, PD	80 μm

**Table 2:** The chemical component of AlSi10Mg powders (wt. %).

Elements	Si	Mg	Mn	Cu	Fe	Ni	Zn	Sn	Ti	Pb	Al
wt. %	10.0	0.40	0.40	0.05	0.50	0.05	0.10	0.05	0.15	0.05	Bal

**Table 3:** The tensile strength of tensile samples with overlapping areas of different sizes.

Test	Overlap Area Width (mm)	Tensile Strength (MPa)
1	0	370
2	0	255
3	0.1	250
4	0.2	325
5	0.3	360
6	0.5	365
7	0.6	340
8	0.7	350
9	0.9	365
10	1.0	355
11	1.1	355
12	1.2	370
13	1.3	340
14	1.4	350
15	1.5	365
